# Supplementary material for: TMEM16K is an interorganelle regulator of endosomal sorting
Source: Nat Commun. 2020 Jul 3;11:3298. doi: 10.1038/s41467-020-17016-8 (PMC7335067; doi:10.1038/s41467-020-17016-8)
Supplement: Supplementary file 1 — Supplementary Information [file 41467_2020_17016_MOESM1_ESM.pdf]

TMEM16K is an interorganelle regulator of endosomal sorting

Maja Petkovic, Juan Oses-Prieto, Alma Burlingame, Lily Yeh Jan, Yuh Nung Jan

Supplementary Information

Content:

- Supplementary Figure 1
- Supplementary Figure 2
- Supplementary Figure 3
- Supplementary Figure 4
- Supplementary Figure 5
- Supplementary Figure 6
- Supplementary Table 1.

- Supplementary Video 1
- Supplementary Video 2
- Supplementary Video 3
- Supplementary Data 1

Supplementary Figure 1

a

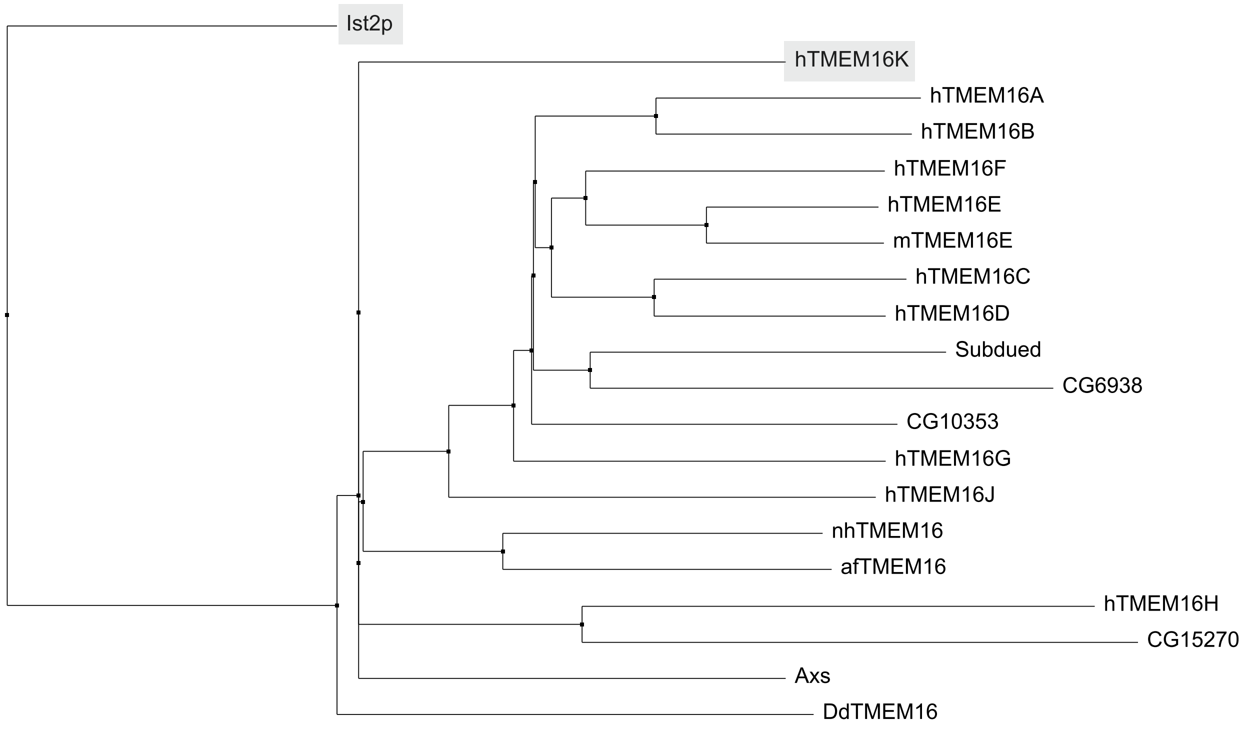

b

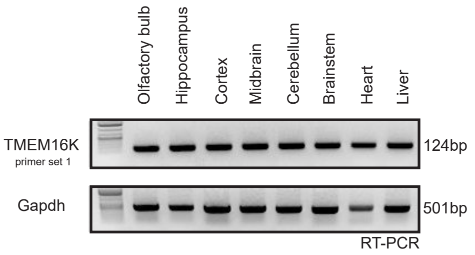

Supplementary Figure 1.a. Phylogenetic tree of TMEM16 family of proteins represented with only homolog in yeast *Saccharomyces cereviseae* (Ist2p), 10 homologs in humans (TMEM16A-H), 5 homologs in Drosophila (Axs, Subdued, CG6938, CG10353, CG15270), only homolog in fungi *Aspergillus fumigatus* (afTMEM16), only homolog in fungi *Nectria haematococca* (nhTMEM16) and only homolog in amoebozoa *Dictyostelium discoideum* (DdTMEM16). Representation was constructed in Jalview based on Neigbourhood Joining algorithm generated from the MUSCLE alignment of the sequences. **b.** RT-PCR from C57BL/6J mouse tissue shows TMEM16K is broadly expressed. RNA was extracted, reverse transcribed and equal amounts of each tissue sample were loaded on agarose gel and revealed with ethidium bromide. Housekeeping gene Gapdh was shown here as a control. DNA Ladder used was GeneRuler 1 kb Plus DNA Ladder from ThermoScientific.

Supplementary Figure 2

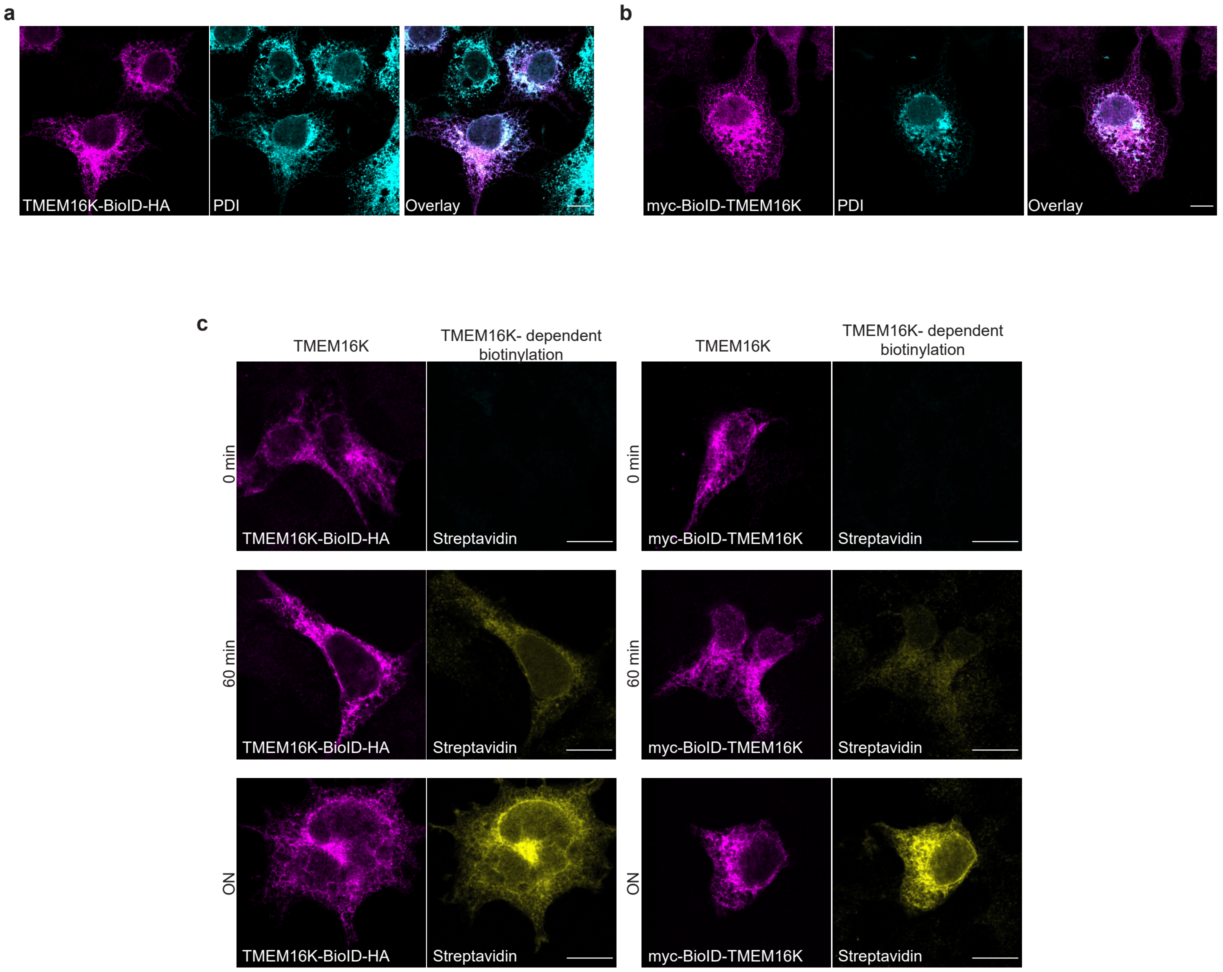

Supplementary Figure 2. a. Immunocytochemistry of TMEM16K tagged C-terminally with proximity biotinylation ezyme revealed with anti-HA antibody (magenta) and ER- marker PDI (cyan). Scale bar 10 μm. **b.** Immunocytochemistry of TMEM16K N-terminally tagged to proximity biotinylation enzyme revealed with anti-myc antibody (magenta) and ER-marker marker PDI (cyan). Scale bar 10 μm. **c.** Immunocytochemistry evaluating retention of biotinylation activity of the BioID tagged to TMEM16K. HEK293 cells were transfected with TMEM16K tagged with biotinylation enzyme constructs (magenta) and 24 hours post-transfection were exposed to no biotin, 60 min or overnight (ON) biotin at 50 μM final concentration. Distribution of TMEM16K-mediated proximity biotinylation was revealed with fluorescently conjugated Streptavidin (yellow). Scale bar 10 μm. Images are represented using pseudocolors suitable for color-blind palette.

Supplementary Figure 3

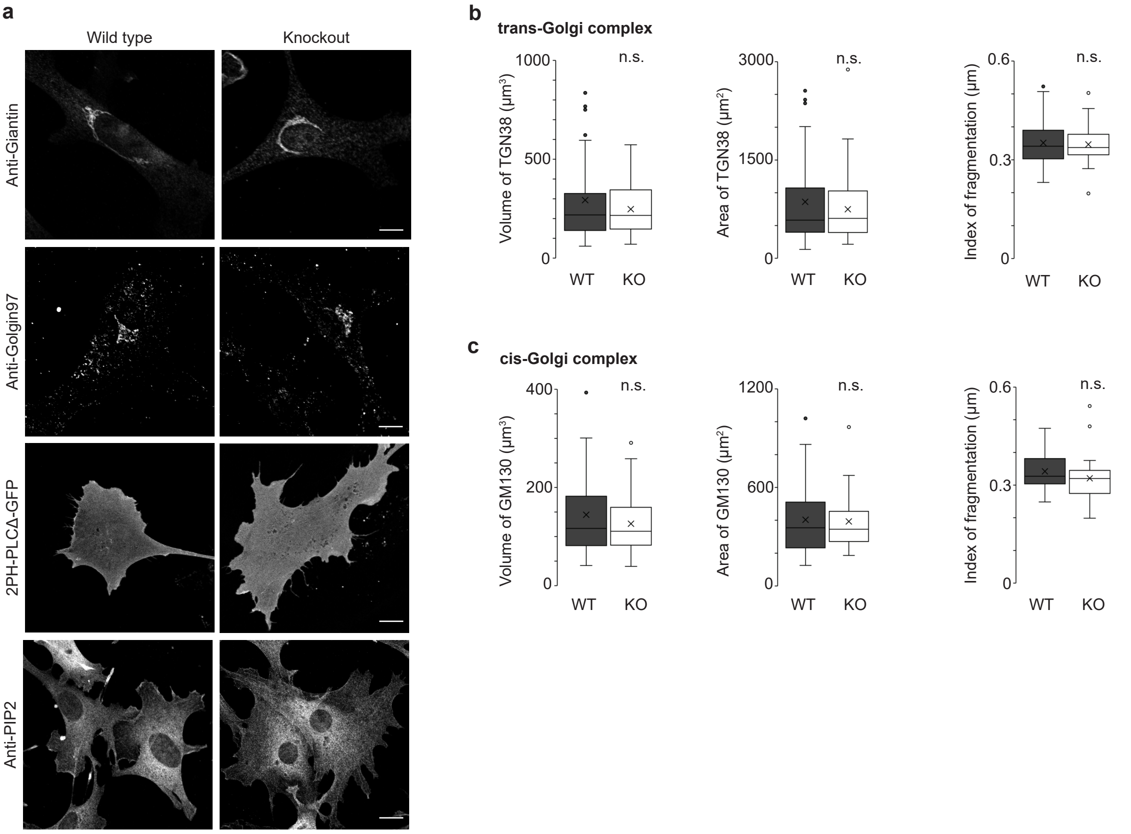

Supplementary Figure 3.a. Cellular markers evaluated in WT or TMEM16K KO cells with immunocytochemistry and confocal microscopy. Giantin and Golgin97 are Golgi markers. 2PH-PLCΔ-GFP and anti-PIP2 antibody are markers of PtdIns(4,5)P_2. Representative images from 2 independent experiments. Scale bar 10 μm. **b.** Evaluation of 3D trans-Golgi complex morphology from 3D reconstructions of endogenous immunolabeling of the trans-Golgi marker TGN38 in the WT (n=99) and TMEM16K KO (n=82) cells. Data obtained from 3 independent experiments Two-tailed Student t-test. Quantification of the volume (p-value=0.76 n.s.), area (p-value=0.89 n.s.) and index of fragmentation, expressed as the TGN38 volume divided by its area for each cell (p-value=0.13 n.s.). **c.** Evaluation of 3D cis-Golgi complex morphology from 3D reconstructions of endogenous immunolabeling of the cis-Golgi marker GM130 in the WT (n=40) and TMEM16K KO (n=36). Data obtained from 3 independent experiments. Two-tailed Student t-test. Quantification of the volume (p-value=0.32 n.s.), area (p-value=0.81 n.s.) and index of fragmentation, expressed as volume of GM130 divided by its area for each cell (p-value=0.17 n.s.).

Supplementary Figure 4

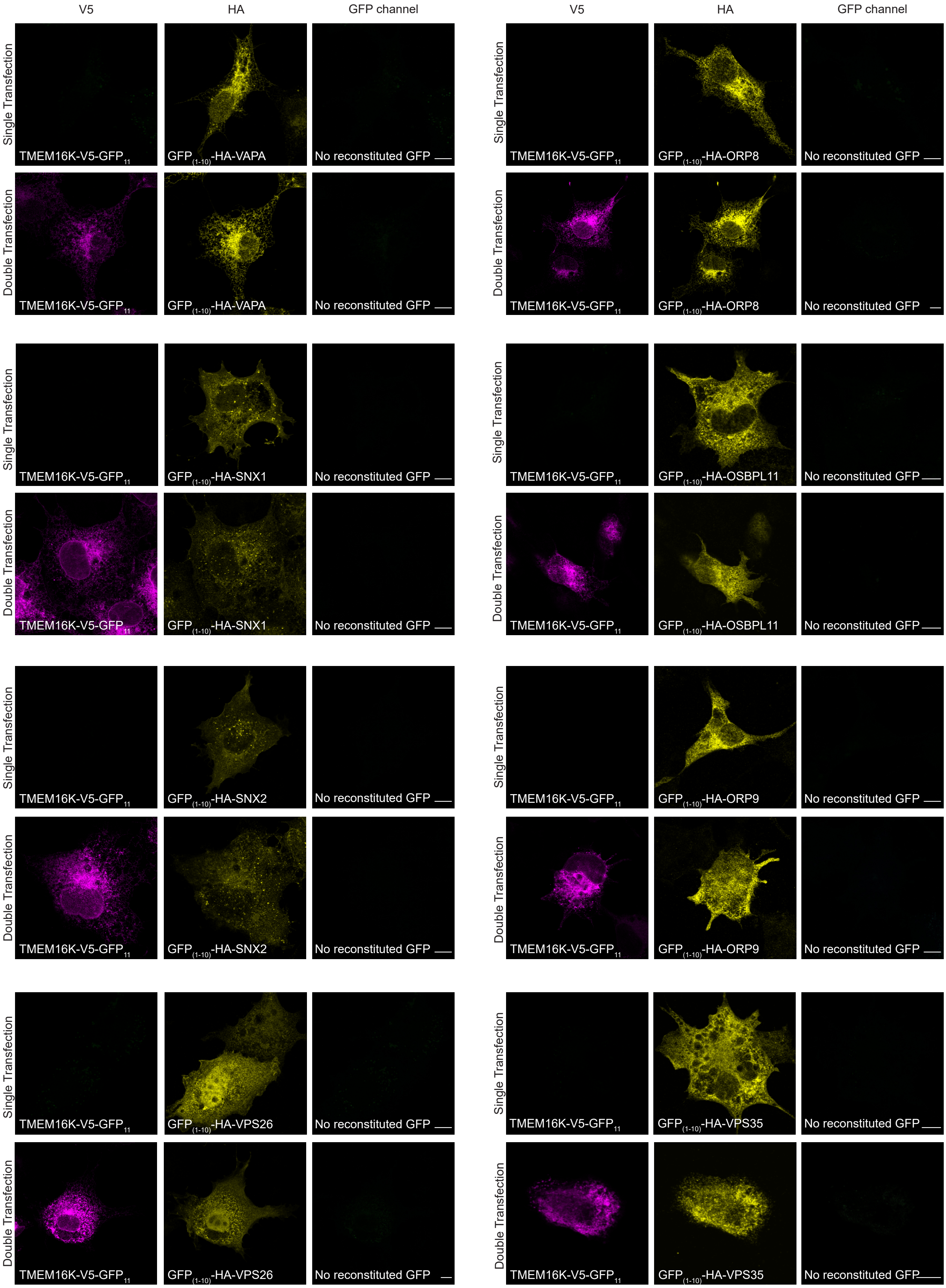

Supplementary Figure 4. Split-GFP reconstitution assay. Immunocytochemistry of single and double transfected COS7 cells with TMEM16K-V5-GFP11 (magenta) and tested proteins tagged with GFP(1-10) (yellow). Images are represented using pseudocolors suitable for color-blind palette. Representative images from 3 independent experiments. Scale bar 10 µm.

Supplementary Figure 5

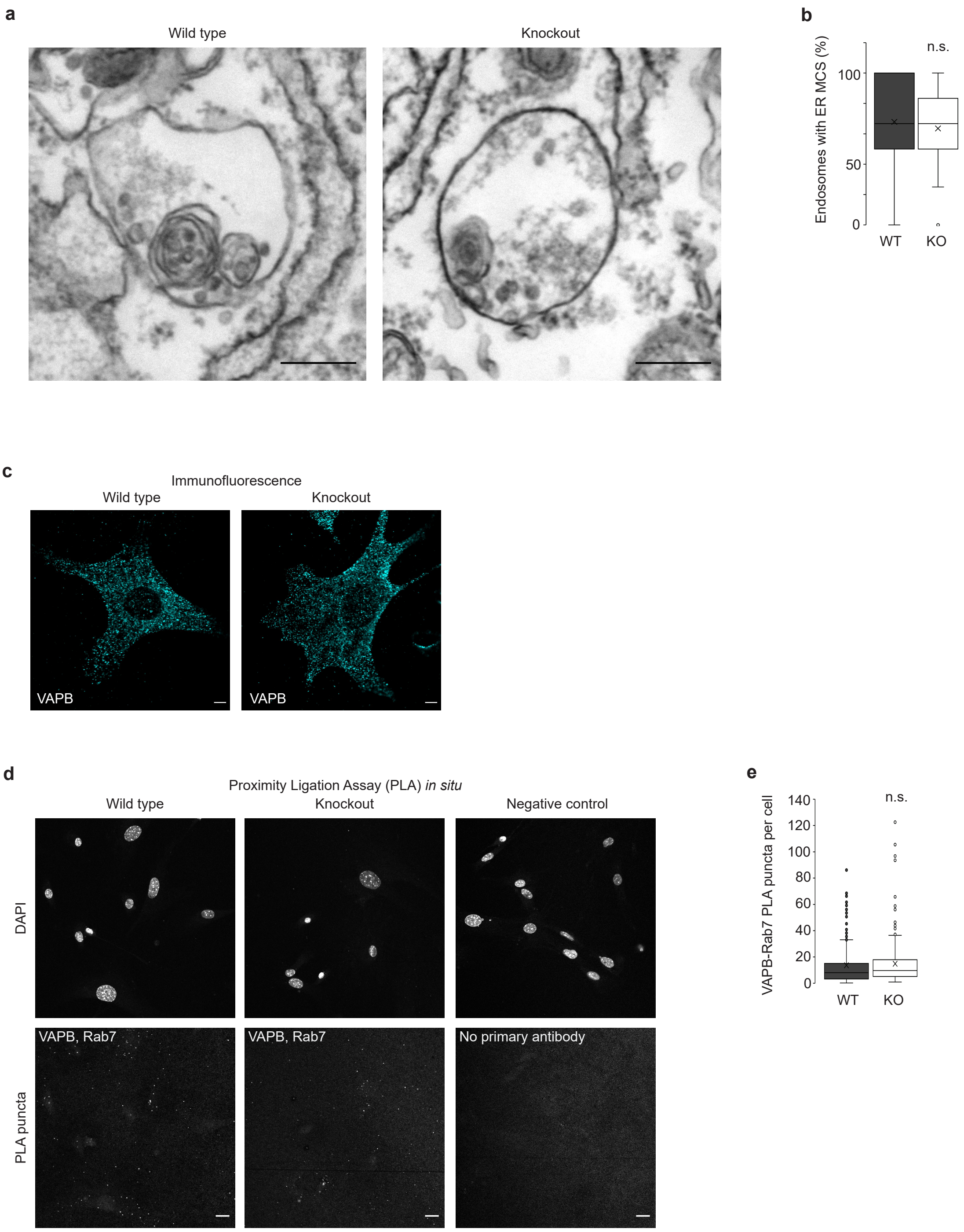

Supplementary Figure 5. a. Electron micrographs showing membrane contact sites between ER and endosomes in the WT and TMEM16K KO primary fibroblasts. Scale bar, 200 nm. **b.** The percentage of endosomes with an ER contact site defined as proximity under 30 nm was quantified. Data are from two independent experiments. (n=68 electron micrographs of WT, 71 micrographs of TMEM16K KO). Two-tailed Student t-test, p-value=0.40 n.s. **c.** Immunofluorescence of WT and TMEM16K KO mouse primary fibroblast to evaluate the VAPB antibody. Scale bar, 5 µm. **d.** Proximity ligation assay (PLA) *in situ* evaluating the extent of ER- endosome contact sites in WT and TMEM16K KO primary fibroblasts. Cells were fixed, permeabilized, labelled with mouse anti-VAPB and rabbit anti-Rab7 antibodies, or with omitted primary antibodies as indicated to confirm the specificity of the observed signal, and subjected to proximity ligation assay *in situ* (PLA). Nuclei were detected with DAPI. Scale bar, 20 µm. **e.** Number of PLA puncta and nuclei from 3 independent experiments was measured, and quantified as PLA puncta per cell. Two-tailed Student t-test, p-value=0.43 n.s. (3 biological replicates, n=231 non-overlapping fields of view with WT cells, 252 with TMEM16K KO cells). Source data are provided as a Source Data file.

Supplementary Figure 6

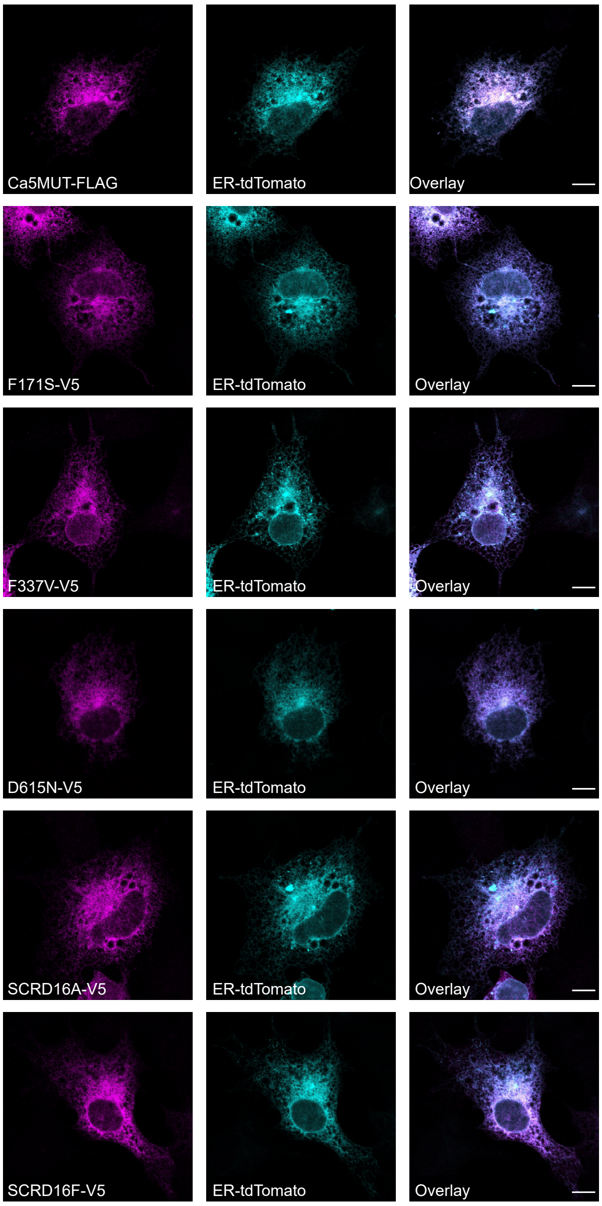

Supplementary Figure 6. Immunocytochemistry to evaluate subcellular localization of TMEM16K mutants to endoplasmic reticulum. COS7 were co-transfected with ER-tdTomato labeling ER (cyan) and TMEM16K mutant constructs tagged with FLAG or V5 tag, which was revealed with anti-FLAG or anti-V5 antibody, respectively (magenta). Scale bar 10 μm. Images are represented using pseudocolors suitable for color-blind palette.

Supplementary Table 2. List of all primers and constructs generated for this study.

TMEM16K genotyping and RT-PCR primers

Primer name	Primer sequence
**Ano10_257998_F**	CACTCCCTCATCCCATTCTTG
**Ano10_257998_R**	AGACGGCCACCTTACCACAG
**Ano10_257998_F and CAS_R1_Term**	TCGTGGTATCGTTATGCGCC
**TMEM16K primer set 1.1**	CATGGCCATCATTGGACTGCCC
**TMEM16K primer set 1.2**	GCACAGCCACGCTTCCACAC
**TMEM16K primer set 2.1**	GCCATGCGGGCCTTCACCTA
**TMEM16K primer set 2.2**	GCCATGCGGGCCTTCACCTA
**Gadph 1.1**	TGGCCCCTCTGGAAAGCTGTG
**Gadph 1.2**	AGTTGGGATAGGGCCTCTCTTGC
**β-actin 1.1**	ATGAGCTGCGTGTGGCCCCTG
**β-actin 1.2**	GACGCAGGATGGCGTGAGGG

Classical subcloning

List of constructs	Backbone	RE to cut backbone	Source of insert	Primers to amplify insert	RE to cut the insert
**myc-BioID-TMEM16K**	pcDNA3.1-myc-BioID (Addgene)	NotI, KpnI	pcDNA3.1-TMEM16K-FLAG (Genscript)	F:gcggcggccgctaagagtgactttatcaacgctggatacttgt<br>R:cccggtacctaggtagcttccttcccatcttcctggt	NotI, KpnI
**TMEM16K-BioID-HA**	BioID-HA (Addgene)	AgeI, BamHI	pcDNA3.1-TMEM16K-FLAG (Genscript)	F:gcgaccggtatgagagtgactttatcaacgctggatacttgt<br>R:cccggatcccaaggtagcttccttcccatcttcctggt	AgeI, BamHI
**mcherry-CAAX**	CIBN-CAAX (Pietro de Camilli)	NheI, AgeI	mcherry cDNA	F:ccgctagcatggtgagcaagggcgaggaggata<br>R: cgaccggtccgcttcgagatctcttgtacagctc	NheI, AgeI
**N16F-GFP**	pEGFP-N1 (Clontech)	NheI, EcoRI	TMEM16F-GFP (Tien et al. 2009)	F:ggcgctagcatgcagatgatgactaggaaggtcctgctgaac<br>R:gcggaattctcctgataagatccaagggctgct	NheI, EcoRI
**N16K-GFP**	pEGFP-N1 (Clontech)	NheI, HindIII	pcDNA3.1-TMEM16K-FLAG (Genscript)	F:gcggctagcaccgccaccatgagagtgacttttat<br>R:ggcaagctttgtctccccaaatagctacgaatgc	NheI, HindIII
**N16A-GFP**	pEGFP-N1 (Clontech)	NheI, EcoRI	TMEM16A-GFP (Tien et al. 2009)	F:gcggctagcccggggttgtggatggggag<br>R:ggcgaattcggccaaccttctcaccaaagta	NheI, EcoRI
**pet15b-His-N16F-GFP**	pet15b (Novagene, Millipore)	NdeI, BlpI	N16F-GFP	F:caagcttccatatgaccgtcagatccgctagcatgc<br>R:gatccggtgctcagcgtccatgccgagagtgatcccg	NdeI, BlpI

**pet15b-His-N16K-GFP**	pet15b (Novagene, Millipore)	NdeI, BlpI	N16K-GFP	F:caagcttccatatgaacaccgccaccatgagagtgact<br>R:gatccggtgctcagctcgtccatgccgagagtgatc	NdeI, BlpI
**pet15b-His-N16A-GFP**	pet15b (Novagene, Millipore)	NdeI, BlpI	N16A-GFP	F:caagcttccatatggttgtggatggggagcgcgag<br>R:gatccggtgctcagcgtccatgccgagagtgatcccg	NdeI, BlpI

Gibson assembly

List of constructs	Backbone	Primers to amplify backbone	Source of insert	Primers to amplify insert
**TMEM16K-V5**	pCDNA3.1-TMEM16K-FLAG (Genescript)	Backbone is amplified in 2 segments splitting the AmpR gene (increases the probability that picked colony is a positive hit) and adding V5 tag with primers<br>Backbone part 1:<br>AmpF: gtaagttggccgcaggttatcactcatgg<br>R1:gtccaagcccagcaaagggttaggaataggcttaccggtagcttccttcccatcttcctggtac<br>Backbone part 2:<br>F2:cctattcctaaccctttgctgggcttggactcaacctagcgctgatcagcctcgactgt<br>AmpR: ccatgagtgataacactgcggccaacttac	V5 tag was added with primers.	
**TMEM16K-V5-GFP11**	pCDNA3.1-TMEM16K-FLAG (Genescript)	Backbone is amplified in 2 segments splitting the AmpR gene.<br>Backbone part 1:<br>AmpF: gtaagttggccgcaggttatcactcatgg<br>R1: caccgcctccgccaccggtagcttccttcccatcttcctggtac<br>Backbone part 2:<br>F2:gctgctgggattacatagcgctgatcagcctcgactgt<br>AmpR: ccatgagtgataacactgcggccaacttac	Synthesized by Integrated DNA Technologies<br>ggtggcggaggcggtggaggtaagcctattcctaaccctttgctgggcttggactcaaccggtggaggaggctcaggtggcggaggatcaggcggtggtggatcacgtgaccacatggtccttcatgagtatgtaaatgctgctgggattacatag	
**GFP$_{(1-10)}$-HA-TMEM16K**	pcDNA3.1-GFP$_{(1-10)}$ (Addgene)	Backbone is amplified in 2 segments splitting the AmpR gene.<br>Backbone part 1:<br>AmpF: gtaagttggccgcaggttatcactcatgg<br>R1:gggtaacctccacctccgcctcctttttcatttggatctttgctcaggactgtttgtg<br>Backbone part 2:<br>F2: gcttaggcggccttaagtttaaaccgc<br>AmpR: ccatgagtgataacactgcggccaacttac	pcDNA3.1-TMEM16K-FLAG (Genscript)	F:gaaaaaggaggcggaggtggaggttacccttacgatgtaccggattacgcaagagtgactttatcaacgctggatacttgt<br>R:gcggtttaaacttaaggccgcctaagctcaggtagcttccttcccatcttcctg
**GFP$_{(1-10)}$-HA-Rab7**			Rab7-tdTomato (UCSF Nikon Imaging Center)	F:gaaaaaggaggcggaggtggaggttacccttacgatgtaccggattacgcaacctctaggaagaaagtgttgctgaaggtt<br>R:gcggtttaaacttaaggccgcctaagctcagcaactgcagctttctgccg
**GFP$_{(1-10)}$-HA-VAPA**			pEGFP-N1-VAPA (Addgene)	F:gaaaaaggaggcggaggtggaggttacccttacgatgtaccggattacgcagcgaaacacgagcaaatcctggtcc<br>R:<br>gcggtttaaacttaaggccgcctaagcctacgagatgaatttccctagaaagaatccaat
**GFP$_{(1-10)}$-HA-SNX1**			SNX1 cDNA (Ewan Reid)	F:gaaaaaggaggcggaggtggaggttacccttacgatgtaccggattacgcagatccggagtcggaaggggc<br>R:gcggtttaaacttaaggccgcctaagctcaggagatggcctttgcctcag

**GFP$_{(1-10)}$-HA-SNX2**			SNX2 cDNA (Marcel Verges)	F:gaaaaaggaggcggaggtggaggttacccttacgatgtaccggattacgcagcggccgagagggaacc<br>R:<br>gcggtttaaacttaaggccgcctaagcctaggcaatggctttggcttcaggtag
**GFP$_{(1-10)}$-HA-VPS26**			VPS26A cDNA (Transomics)	F:gaaaaaggaggcggaggtggaggttacccttacgatgtaccggattacgcaatgagttttcttggaggctttttggtcca<br>R:gcggtttaaacttaaggccgcctaagctcacatttcaggctgttcggcagatgc
**GFP$_{(1-10)}$-HA-ORP8**			ORP8 cDNA (Francesca Giordano)	F:gaaaaaggaggcggaggtggaggttacccttacgatgtaccggattacgcagagggaggtttggcagatggagaac<br>R:gcggtttaaacttaaggccgcctaagcctacttgaacatgaagtttattatgacttgaag
**GFP$_{(1-10)}$-HA-OSBPL11**			OSBPL11 cDNA (Transomics)	F:gaaaaaggaggcggaggtggaggttacccttacgatgtaccggattacgcacaggggggtgaaccagtgtcc<br>R:gcggtttaaacttaaggccgcctaagctcactctgctggttgtgttgttggaattatttt
**GFP$_{(1-10)}$-HA-VPS35**			VPS35 cDNA (Marcel Verges)	F:gaaaaaggaggcggaggtggaggttacccttacgatgtaccggattacgcacctacaacacagcagtcccctcag<br>R:gcggtttaaacttaaggccgcctaagcttaaaggatgagaccttcataaattggccc
**GFP$_{(1-10)}$-HA-ORP9**	GFP$_{(1-10)}$-HA-TMEM16K	Backbone is amplified in 2 segments splitting the AmpR gene.<br>Backbone part 1:<br>AmpF: gtaagttggccgcaggttatcactcatgg<br>R1-ORP9:<br>gcctcctcctccgccgcctgcgtaatccggtacatcgtaagggtaacc<br>Backbone part 2:<br>F2-ORP9:gctgccaagcattaggcttaggcggccttaagtttaaaccgc<br>AmpR: ccatgagtgataacactgcggccaacttac	ORP9 cDNA (Transomics)	F:ggcggcggaggaggaggcatggtagaatcaattaaacactgcattgtgttgc<br>R:cttaaggccgcctaagcctaatgcttggcagcaccaagacg
**mClover3-TMEM16K**	pSBtet-RB (Addgene)	Backbone is amplified in 2 segments splitting the AmpR gene.<br>Backbone part 1:<br>AmpF:gtaagttggccgagtgttatcactcatgg<br>R1:gcccttctcaccatggtggcctcagaggcctttcg<br>Backbone part 2:<br>F2:gaaggagctacctgaggctgtcaggccaagcttc<br>AmpR:ccatgagtgatacactgcggccacttac	pKanCMV-mClover3-mRuby3 (Addgene)<br>pcDNA3.1-TMEM16K-FLAG (Genscript)	Insert 1 (mClover3):<br>F1:ctctgaggccaccatggtgagcaagggcgaggagc<br>R1:gataaagtcactctcattcttcgcctttgctcaccatcgg<br>Insert 2 (TMEM16K):<br>2F:gagcaaaggcgaagaaatagagtgactttatcaacgctggatcttgtgagag<br>2R:cttggcctgacaggctcaggtagcttccttcccatctcctg
**TMEM16K-V5-mNeonGreen**	TMEM16K-V5-GFP11	Backbone is amplified in 2 segments splitting the AmpR gene.<br>Backbone part 1:<br>AmpF:gtaagttggccgagtgttatcactcatgg<br>NG-R1: GCCCTTGCTCACCATTGAtccaccaccgccTGAtcc<br>Backbone part 2:<br>AmpR:ccatgagtgatacactgcggccacttac<br>NG-F2: GACGAGCTGTACAAGtagcgctgatcagcctcgactgt	mNeonGreen-mRuby2-FRET-10 (UCSF Nikon Imaging Center Library)	Insert (mNeonGreen)<br>NG-F1:<br>CAggcggtggtggaTCAATGGTGAGCAAGGGCGAGGAG<br>NG-R2:<br>ggctgatcagcgctaCTTGTACAGCTCGTCCATGCCCATC
**SCRD16A-V5**	TMEM16K-V5	Backbone is amplified in 2 segments splitting the AmpR gene.	Synthesized by Integrated DNA Technologies	

<table>
<tr><td></td><td></td><td>Backbone part 1:
R1:gccgtaaacttcatccatgatctcaatcacaacggcataaacaatgctgg
AmpF: gtaagttggccgcaggttatcactcatgg
Backbone part 2:
F2: gccttcctgctcaagttcctgaattgcttcgcctcactcttctac
AmpR: ccatgagtgataacactgcggccaacttac</td><td colspan="2">gtgattgagatcatggatgaagtttacggctgcattgccaggtggctcaccaagattgaggtcccaaa
gacagagaagagctttgaggagaggctaaccttcaaggccttcctgctcaagttcctgaattgcttc</td></tr>
<tr><td>SCRD-16F-V5</td><td>TMEM16-V5</td><td>Backbone is amplified in 2 segments splitting the AmpR gene.
Backbone part 1:
R1:ctcgtagatcgtgttcatgatctcaatcacaacggcataaacaatgctgg
AmpF: gtaagttggccgcaggttatcactcatg
Backbone part 2:
F2: gatgttcttgttccag ttcctgaattgcttcgcctcactcttctac
AmpR: ccatgagtgataacactgcggccaacttac</td><td colspan="2">Synthesized by Integrated DNA Technologies
gtgattgagatcatgaacacgatctacgagaaggtggccatcatgatcaccaacttcgagctccca
aggacccagacggattatgagaacagcctgaccatgaagatgttcttgttccagttcctgaattgcttc</td></tr>
<tr><td>SCRD16A-V5-GFP11</td><td rowspan="5">TMEM16K-V5-GFP11</td><td rowspan="5">Backbone is amplified in 2 segments splitting the AmpR gene.
Backbone part 1:
R1:
CTCTCACAAGTATCCAGCGTTGATAAAGTCACTCTCAT
AmpF: gtaagttggccgcaggttatcactcatg
Backbone part 2:
F2: CCAGGAAGATGGGAAGGAAGCTACC
AmpR: ccatgagtgataacactgcggccaacttac</td><td>SCRD16A-V5</td><td rowspan="5">All respective inserts were amplified with following primers
K-F:
ATGAGAGTGACTTTATCAACGCTGGATACTTGTG
AGAG
K-R: GGTAGCTTCCTTCCCATCTTCCTGG</td></tr>
<tr><td>SCRD16F-V5-GFP11</td><td>SCRD16F-V5</td></tr>
<tr><td>F171S-V5-GFP11</td><td>F171S-V5</td></tr>
<tr><td>F337V-V5-GFP11</td><td>F337-V5</td></tr>
<tr><td>D615N-V5-GFP11</td><td>D615N-V5</td></tr>
<tr><td>deltaN16K-V5-GFP11</td><td>TMEM16K-V5-GFP11</td><td>Backbone is amplified in 2 segments splitting the AmpR gene, and removing the N-terminal domain of TMEM16K
Backbone part 1:
AmpF: gtaagttggccgcaggttatcactcatgg
dNtR:
GTCATGCAGCGGGAACACcatgccagcttgggtctccctatag
Backbone part 2:
dNtF:gacccaagctggcatgGTGTTCCCGCTGCATGACACTGA
AmpR: ccatgagtgataacactgcggccaacttac</td><td colspan="2"></td></tr>
</table>

Site-directed mutagenesis

List of constructs	Backbone	Site-directed mutagenesis primers	Comments
**Ca5MUT-FLAG (TMEM16K-E448Q/D497N/E500Q/E529Q/D533N-FLAG)**	pCDNA3.1.-TMEM16K-FLAG (Genscript)	448-F: CTGAACCAAGTCGTAcAATCTCTTCTTCCTT<br>448-R: AAGGAAGAAGAGATTgTACGACTTGGTTCAG<br>497-500-F: CTGGGAACCTTTGATaATTACCTGcAGTTGTTCCTGCAGT<br>497-500-R: ACTGCAGGAACAACTgCAGGTAATtATCAAAGGTTCCCAG<br>529-533-F: GTTAAATAACTTCACTcAAGTCAACTCAaATGCCTTGAAAATGTG<br>529-533-R: CACATTTTCAAGGCATtTGAGTTGACTTgAGTGAAGTTATTTAAC	All 5 mutations were introduced in one multiplex-site-directed mutagenesis reaction.
**F171S-V5**	TMEM16K-V5	F171S.F: GGCATCGTGACCCAAGTGTcCCCGCTGCAT<br>F171S.R: ATGCAGCGGGgACACTTGGGTCACGATGCC	
**F337-V5**	TMEM16K-V5	F337V.F: TATGTCATGATGATCTACgTTGACATGGAGGACTGGG<br>F337V.R: CCCAGTCCTCCATGTCAAcGTAGATCATCATGACATA	
**D615N-V5**	TMEM16K-V5	D615N.F: TCGCATTTGCCATCCCTaATAAACCACGGCACATC<br>D615N.R: GATGTGCCGTGGTTTATtAGGGATGGCAAATGCGA	
**GFP$_{(1-10)}$-HA-Rab7 T22N**	GFP(1-10)-HA-Rab7	T22N-R: CTGGTTCATGAGTGAgtTCTTCCCGACTCCAGA<br>T22N-F: CTGGAGTCGGGAAGAacTCACTCATGAACCAGT	
**GFP$_{(1-10)}$-HA-Rab7 Q67L**	GFP(1-10)-HA-Rab7	Q67L-R: GAGACTGGAACCGTTCCaGTCCTGCTGTGTCCC<br>Q67L-F: GGGACACAGCAGGACtGGAACGGTTCCAGTCT	
