# Supplementary material for: TMEM16K is an interorganelle regulator of endosomal sorting
Source: Nat Commun. 2020 Jul 3;11:3298. doi: 10.1038/s41467-020-17016-8 (PMC7335067; doi:10.1038/s41467-020-17016-8)
Supplement: Supplementary file 3 — Description of Additional Supplementary Files [file 41467_2020_17016_MOESM3_ESM.pdf]

Description of Additional Supplementary Files

File Name: Supplementary Data 1
Description: Candidate list emerged from the proteomic mapping of protein complexes surrounding TMEM16K via in situ BioID-catalyzed biotin labeling

File Name: Supplementary Movie 1
Description: Representative hindlimb clasping of neuron specific TMEM16K WT and KO animal at 24 months of age.

File Name: Supplementary Movie 2
Description: Live imaging of COS7 cells transfected with mClover3- TMEM16K (pseudocolored in magenta) and ER-tdTomato (pseudocolored in cyan). Video presents overlay of the two channels.

File Name: Supplementary Movie 3
Description: Live imaging of COS7 cell transfected with TMEM17K-V5- mNeonGreen (pseudocolored in magenta), tdTomato-Rab7 (pseudocolored in yellow) and EGF-Alexa647 incubated for 45 min (pseudocolored in cyan) on spinning disk confocal. Video presents overlay of the three channels.
