# Supplementary material for: TMEM16K is an interorganelle regulator of endosomal sorting
Source: Nat Commun. 2020 Jul 3;11:3298. doi: 10.1038/s41467-020-17016-8 (PMC7335067; doi:10.1038/s41467-020-17016-8)
Supplement: Supplementary file 8 — Reporting Summary [file 41467_2020_17016_MOESM8_ESM.pdf]

nature research

Corresponding author(s): Yuh Nung Jan, Maja Petkovic

Last updated by author(s): 05/30/2020

Reporting Summary

Nature Research wishes to improve the reproducibility of the work that we publish. This form provides structure for consistency and transparency in reporting. For further information on Nature Research policies, see Authors & Referees and the Editorial Policy Checklist .

Statistics

For all statistical analyses, confirm that the following items are present in the figure legend, table legend, main text, or Methods section.

n/a	Confirmed	
☐	☒	The exact sample size (*n*) for each experimental group/condition, given as a discrete number and unit of measurement
☐	☒	A statement on whether measurements were taken from distinct samples or whether the same sample was measured repeatedly
☐	☒	The statistical test(s) used AND whether they are one- or two-sided<br>*Only common tests should be described solely by name; describe more complex techniques in the Methods section.*
☐	☒	A description of all covariates tested
☐	☒	A description of any assumptions or corrections, such as tests of normality and adjustment for multiple comparisons
☐	☒	A full description of the statistical parameters including central tendency (e.g. means) or other basic estimates (e.g. regression coefficient) AND variation (e.g. standard deviation) or associated estimates of uncertainty (e.g. confidence intervals)
☐	☒	For null hypothesis testing, the test statistic (e.g. *F*, *t*, *r*) with confidence intervals, effect sizes, degrees of freedom and *P* value noted<br>*Give P values as exact values whenever suitable.*
☒	☐	For Bayesian analysis, information on the choice of priors and Markov chain Monte Carlo settings
☒	☐	For hierarchical and complex designs, identification of the appropriate level for tests and full reporting of outcomes
☒	☐	Estimates of effect sizes (e.g. Cohen's *d*, Pearson's *r*), indicating how they were calculated

Our web collection on statistics for biologists contains articles on many of the points above.

Software and code

Policy information about availability of computer code

Data collection	No software was used to collect the data.
Data analysis	We have used Protein Prospector v 6.2.1 (UCSF), Cytoscape 3.7.1, and Fiji Software 1.52 (ImageJ, NIH) which are all open source software platforms. We used PAVA in-house software referenced in the study. We have used commercial Imaris 9.5 Software (Oxford Instruments) for 3D image reconstruction and quantification. We used open source Pymol 2.1 software to visualize relevant residues on published crystal structure of TMEM16K.

For manuscripts utilizing custom algorithms or software that are central to the research but not yet described in published literature, software must be made available to editors/reviewers. We strongly encourage code deposition in a community repository (e.g. GitHub). See the Nature Research guidelines for submitting code & software for further information.

Data

Policy information about availability of data

All manuscripts must include a data availability statement. This statement should provide the following information, where applicable:

- Accession codes, unique identifiers, or web links for publicly available datasets
- A list of figures that have associated raw data
- A description of any restrictions on data availability

The data that support the findings of this study are available from the corresponding author upon reasonable request. The source data underlying Figs 1b, 1c, 1e, 1h, 3a, 3b, 4a-f, 4h6g, 7d, 8e,Supplementary Figs 3b ,3c, Supplementary Figs 5b, 5e are provided as a Source Data file. Our proteomics datasets are available via ProteomeXchange with identifier PXD018990. We have used publicly available String and SwissProt databases.

Field-specific reporting

Please select the one below that is the best fit for your research. If you are not sure, read the appropriate sections before making your selection.

- [x] Life sciences
- [ ] Behavioural & social sciences
- [ ] Ecological, evolutionary & environmental sciences

For a reference copy of the document with all sections, see nature.com/documents/nr-reporting-summary-flat.pdf

Life sciences study design

All studies must disclose on these points even when the disclosure is negative.

Sample size

No sample size calculations were performed. Sufficient sample size was determined by comparison with similar published experiments in literature and our ability and technical possibility to maximize sample sizes in our experiments.

Mouse knockout analyses in literature reported to use min. 2-3 animals for histology and min. 5-6 animals for behavioral studies as done in Thomas et al. Human Molecular Genetics 2006, PMID: 16772330; Caillol et al. Eur J Neuroscience 2012, PMID: 22642323; Furrer et al. Human Molecular Genetics 2013. PMID: 23197655. Analysis of the endosomal retrograde transport as done in Hoyer et al. Cell 2018, PMID: 30220460 used cca 30 cells per condition, as well as Dong et al. Cell, 2016, PMID: 27419871, where they used cca 55-57 cells per condition. We performed RUSH assay similarly and with same constructs as done in Chen et al. J. Cell Biol. 2017, PMID: 28978644, where they used cca 12 cells per condition. 3D morphology of Golgi complex was analyzed similar as it was done by Thayer at al. PNAS, 2013, PMID: 23297202, where they used between 10-20 cells per condition. We performed electron microscopy analysis of ER-endosome membrane contact sites as done in Kilpatrick et al. Cell Reports, 2017, PMID: 28199837, which used for various EM analysis min of 50 cells per condition. Proximity ligation assay done in Lim et al. Nature Cell Biology, 2019, PMID: 31548609, used cca 60 non-overlaping fields of view per condition.

Data exclusions

As listed in Methods, in analysis of proteomic mapping of TMEM16K all proteins that had more than one peptide detected in control conditions were considered background. Next, only those proteins that were at least 3-fold enriched compared to control condition were considered potential interactors, and used for generating protein-protein interactions network, functional enrichment and identification of major clusters. This is standard procedure when dealing with datasets and we predefined stringency level for defining what we consider specific hits. In addition, all the raw data are available via ProteomeXchange.

During image acquisition, rarely we found cells that looked obviously unhealthy and they were not imaged. Rational is that imaging individual cells that are obviously dying or unhealthy due to reasons like overexpression, are not providing biological information, and were equally applied irrespective of the genotype or the condition. See randomization.

No other exclusion was done.

Replication

All experiments included in this study were done in biological replicates, as listed in each figure legend for each experiment. All attempts at replication were successful.

Randomization

Mice cohorts of wild type and knockout animals were genotyped as newborn pups, assigned a random numeric code and after weaning randomly housed based on their genotype with up to 5 same sex animals per cage. Analysis of electron micrographs was done in random order irrespective of the genotype. Imaging of cellular assays on wild type and knockout MEF was done in random order.

Blinding

As listed in Methods, behavior analyses were done blinded of the mice genotype, with each mice tracked during the analysis with a random numeric code. Same holds true for neuromuscular junction analysis, as genotypes were reveled after the image acquisition and measurements. Endosomes and endoplasmic reticulum were identified in electron microscopy micrographs blinded by the cell genotype.

Reporting for specific materials, systems and methods

We require information from authors about some types of materials, experimental systems and methods used in many studies. Here, indicate whether each material, system or method listed is relevant to your study. If you are not sure if a list item applies to your research, read the appropriate section before selecting a response.

Materials & experimental systems

n/a	Involved in the study	
[ ]	[x]	Antibodies
[ ]	[x]	Eukaryotic cell lines
[x]	[ ]	Palaeontology
[ ]	[x]	Animals and other organisms
[x]	[ ]	Human research participants
[x]	[ ]	Clinical data

Methods

n/a	Involved in the study	
[x]	[ ]	ChIP-seq
[x]	[ ]	Flow cytometry
[x]	[ ]	MRI-based neuroimaging

Antibodies

Antibodies used

We used following primary antibodies: rabbit anti-V5 (Cell Signalling tech, #13202, 1/1000), mouse anti-V5 mouse (Invitrogen, # R960-25, 1/1000), mouse anti-FLAG (Sigma, # F1804, 1/1000), rabbit anti-GM130 (Abcam, # ab52649, 1/500), rabbit anti-Rab7 (Cell Signalling tech # 9367, 1/500), mouse anti-myc (kind gift from J. Michael Bishop, clone 9E10, 1/1000), rat anti-HA (Roche, #

11867431001, 1/1000), mouse anti-GFP (Roche, # 11814460001, 1/1000), rabbit anti-calreticulin (Abcam, # ab2907, 1/1000), mouse anti-PDI (Abcam, # ab2792, 1/500), rabbit anti-giantin (kind gift from Marc Von Zastrow, 1/1500), mouse anti-Golgin97 (Molecular probes, # A21270, 1/200), rabbit anti-mCherry (Abcam, # ab167453, 1/1000), chicken anti-mCherry (Novus Biologicals, #NBP2-25158, 1/200), mouse anti-EEA1 (BD Biosciences, #610456, 1/200), mouse anti-PIP2 (Abcam, # ab11039, 1/250), rabbit anti-TGN38 (Novus Biologicals, #NBP1-03495SS, 1/100), and mouse anti-VAPB (R&D systems, #MAB7329-SP, 1/100 for PLA in situ). Secondary antibodies (used at 1/400) and Streptavidin conjugated with Alexa Fluor 647 (# 016-600-084, 1/2000) were purchased from Jackson laboratories or Invitrogen.

Validation

All antibodies used are commercially available.

-rabbit anti-V5 (Cell Signalling tech, #13202, 1/1000): Recommended by manufacturer for WB, IF, IP with 76 citation;

-mouse anti-V5 mouse (Invitrogen, # R960-25, 1/1000): Recommended by manufacturer for WB, IF, IP, ICC with >90 citations for each of the applications;

-mouse anti-FLAG (Sigma, # F1804, 1/1000): Recommended by manufacturer for WB, IF, IP, ICC with >4747 citations;

-rabbit anti-GM130 (Abcam, # ab52649, 1/500): Recommended by manufacturer for ICC/IF with 121 citations, Labome database reports Abcam GM130 antibody (Abcam, ab52649) was used in western blot knockout validation on mouse samples (fig 8). Cell Death Dis (2017);

-rabbit anti-Rab7 (Cell Signalling tech # 9367, 1/500): Recommended by manufacturer for WB, IF, IP for human, mouse, rat and monkey protein with 150 citations, Labome databse reports Cell Signaling Technology RAB7A antibody (Cell Signaling, 9367) was used in immunocytochemistry knockout validation on mouse samples (fig 1), in western blot knockout validation on mouse samples (fig 1) and in flow cytometry on mouse samples (fig 1). Autophagy (2013); Cell Signaling Technology RAB7A antibody (Cell Signaling, 9367) was used in proximity ligation assay on human samples at 1:100 (fig 5b, 5f). Cell Rep (2018)

-mouse anti-myc (kind gift from J. Michael Bishop, clone 9E10, 1/1000): Deposited by J. Michael Bishop to Developmental Studies Hybridoma Bank, which reports recommended use in WB, IP IF, ICC, flow cytometry and ELISA with 139 citations, Labome database reports use of J. Michael Bishop, clone 9E10, c-myc antibody in immunofluorescence in these papers: 24567331, 21145886, 20335479, 2593074, 24273166, 23959653, 24423648, 25136332, 25253864, 24999027, 24746823, 24899700, 26234537, 29661855, 26994136 (PubMed IDs).

-rat anti-HA (Roche, # 11867431001, 1/1000): Recommended by manufacturer for WB, IP, ICC, ELISA with 3 citations

-mouse anti-GFP (Roche, # 11814460001, 1/1000): Recommended by manufacturer for WB, IP, IF with 11 citations

-rabbit anti-calreticulin (Abcam, # ab2907, 1/1000): Recommended by manufacturer for WB, IP, IF, ICC for Mouse, Rat, Rabbit, Dog, Human, Drosophila melanogaster, Non human primates, with 126 citations

-mouse anti-PDI (Abcam, # ab2792, 1/500): Recommended by manufacturer for WB, IF, ICC for Mouse, Human, Rat, African Monkey, with 90 citations

-rabbit anti-giantin (kind gift from Marc Von Zastrow, 1/1500) is made by Covance Cat# PRB-114C-200; RRID: AB_291560, Recommended by manufacturer for IF, ICC for Mouse, Human, Rat, Monkey, with 7 citations

-mouse anti-Golgin97 (Molecular probes, # A21270, 1/200): Recommended by manufacturer for IF, ICC for Mouse, Human, Non-human primates, with >30 citations for each application. Labome database reports golgin-97 antibody (Molecular Probes, A21270) was used in immunocytochemistry knockout validation on human samples (fig 9). Mol Biol Cell (2004)

-rabbit anti-mCherry (Abcam, # ab167453, 1/1000): Recommended by manufacturer for IF, ICC, WB, with 144 citations

-chicken anti-mCherry (Novus Biologicals, #NBP2-25158, 1/200): Recommended by manufacturer for IF, ICC, WB, with 19 citations

-mouse anti-EEA1 (BD Biosciences, #610456, 1/200): Recommended by manufacturer for IF, ICC, WB for Rat, Dog, Human, Chicken with 19 citations. Labome database reports BD Biosciences EEA1 antibody (BD Pharmingen, 610457) was used in western blot on mouse samples at 1:1000 (fig 7e). Nat Cell Biol (2018), in western blot on mouse samples (tbl 1). Neuron (2017), in immunocytochemistry on mouse samples at 1:500. Neuroscience (2015)

-mouse anti-PIP2 (Abcam, # ab11039, 1/250): Recommended by manufacturer for IF, ICC, IP, with 17 citations

-rabbit anti-TGN38 (Novus Biologicals, #NBP1-03495SS, 1/100): Recommended by manufacturer for WB, ICC/IF, IBC for Human, Mouse, Rat, Primate, with 9 citations.

-mouse anti-VAPB (R&D systems, #MAB7329-SP, 1/100 for IF and PLA in situ): Recommended by manufacturer for WB , IHC and ELISA. We have performed immunofluorescence on the WT and TMEM16K KO primary fibroblasts at the same concentration and fixation/permeabilization as used in PLA assay to evaluate the antibody.

Eukaryotic cell lines

Policy information about cell lines

Cell line source(s)

HEK293T, COS7 and U2OS cells. These cell line were obtained from our lab stock, and are frequently used in our lab for multitude of studies. Jan lab acquired cells at ATTC.

Authentication

Cell lines used had expected morphology, media requirements, and electrophysiological properties as expected. We have not performed additional authentication studies for these cell lines.

Mycoplasma contamination

Cell lines were not tested for mycoplasma contamination during the course of our study.

Commonly misidentified lines
(See ICLAC register)

No cells from the ICLAC register of commonly misidentified lines were used in this study.

Animals and other organisms

Policy information about studies involving animals; ARRIVE guidelines recommended for reporting animal research

Laboratory animals	We have used transgenic mice lines in our study: We obtained TMEM16K conditional knockout mice (Ano10tm1a(EUCOMM) Wtsi)) generated by the International Mouse Knockout Consortium and ordered from EMMA (EMMA ID: 08927). Ubiquitous TMEM16K knockout mice were generated by crossing with the actin-driven Cre line, while crossing with nestin-Cre line generated neuron specific TMEM16K knockouts. All procedures performed have been approved by the UCSF IACUC. We used both male and female mice for the aging cohort. They were housed 5 mice of the same sex but random genotype per cage, with food and water available ad libidum.
Wild animals	No wild animals were used in this study.
Field-collected samples	No field collected samples were used.
Ethics oversight	Animal care is provided and controlled by The Laboratory Animal Resource Center (LARC). All procedures performed have been approved by the UCSF Institutional Animal Care Use Committee (IACUC). UCSF has accreditation form the Association for Assessment and Accreditation of Laboratory Animal Care (AAALAC).

Note that full information on the approval of the study protocol must also be provided in the manuscript.
